# Corn Oil Lowers Plasma Cholesterol Compared with Coconut Oil in Adults with Above-Desirable Levels of Cholesterol in a Randomized Crossover Trial

**DOI:** 10.1093/jn/nxy156

**Published:** 2018-09-10

**Authors:** Kevin C Maki, Wendy Hasse, Mary R Dicklin, Marjorie Bell, Mary A Buggia, Martha E Cassens, Fulya Eren

**Affiliations:** 1 Midwest Biomedical Research: Center for Metabolic and Cardiovascular Health, Glen Ellyn, IL; 2 Great Lakes Clinical Trials, Chicago, IL; 3 MB Clinical Research, Boca Raton, FL; 4 ACH Food Companies Inc., Oakbrook Terrace, IL

**Keywords:** corn oil, coconut oil, lipoprotein lipids, inflammation, insulin sensitivity

## Abstract

**Background:**

Few trials have examined the effects of coconut oil consumption in comparison with polyunsaturated fatty acid–rich oils such as corn oil.

**Objective:**

This trial assessed the effects of consuming foods made with corn oil compared with coconut oil on lipids, glucose homeostasis, and inflammation.

**Methods:**

This was a preliminary randomized crossover study of men (*n* = 12) and women (*n* = 13) with a mean age of 45.2 y, mean body mass index (in kg/m^2^) of 27.7, fasting LDL cholesterol ≥115 mg/dL and <190 mg/dL, and triglycerides (TGs) ≤375 mg/dL. Subjects consumed muffins and rolls providing 4 tablespoons (∼54 g) per day of corn oil or coconut oil as part of their habitual diets for 4 wk, with a 3-wk washout between conditions. Fasting plasma lipids and high-sensitivity C-reactive protein (hs-CRP) and glucose metabolism were assessed via an intravenous glucose tolerance test at baseline and 15 and 29 d of treatment. Responses were compared between treatments by ANCOVA.

**Results:**

Median baseline concentrations of LDL cholesterol, non-HDL cholesterol, total cholesterol (total-C), HDL cholesterol, total-C:HDL cholesterol, and TGs were 123, 144, 188, 46.0, 4.21, and 92.5 mg/dL, respectively. Changes from baseline for corn oil and coconut oil conditions, respectively, were: LDL cholesterol (primary outcome; −2.7% compared with +4.6%), non-HDL cholesterol (−3.0% compared with +5.8%), total-C (−0.5% compared with +7.1%), HDL cholesterol (+5.4% compared with +6.5%), total-C:HDL cholesterol (−4.3% compared with −3.3%), and TGs (−2.1% compared with +6.0%). Non-HDL cholesterol responses were significantly different between corn and coconut oil conditions (*P* = 0.034); differences between conditions in total-C and LDL cholesterol approached significance (both *P* = 0.06). Responses for hs-CRP and carbohydrate homeostasis parameters did not differ significantly between diet conditions.

**Conclusions:**

When incorporated into the habitual diet, consumption of foods providing ∼54 g of corn oil/d produced a more favorable plasma lipid profile than did coconut oil in adults with elevated cholesterol. This trial was registered at clinicaltrials.gov as NCT03202654.

## Introduction

Corn oil is a commonly consumed food oil in the United States (proprietary data provided by IRI, Chicago, IL). Compared with some other edible oils, it is high in PUFAs (50–55% of its fatty acids) and rich in phytosterols (534 mg/54 g [4 tablespoons] of oil), both of which reduce atherogenic cholesterol levels (1–4). A qualified health claim exists for corn oil and corn oil–containing products and coronary heart disease, based on their LDL cholesterol–lowering effects (5). The effects of corn oil consumption on lipoprotein lipids have been compared with canola oil (6), with sunflower/olive oil combinations (7), and, in a controlled feeding study, with extra-virgin olive oil (EVOO) (8, 9). Lichtenstein et al. reported that total cholesterol (total-C) was decreased significantly more with canola oil (12%) and corn oil (13%) consumption than with olive oil consumption (7%), but LDL cholesterol responses did not differ among oils (reductions of 16%, 17%, and 13%, respectively) (6). Wagner et al. compared the effects of consumption of a MUFA-rich plant oil mixture (olive/sunflower oil) with consumption of corn oil (PUFA-rich), and demonstrated that LDL cholesterol and VLDL cholesterol were reduced only with the corn oil diet (7). In the controlled feeding study comparing corn oil with EVOO, corn oil was shown to reduce LDL cholesterol by 10.9% compared with a 3.5% reduction with EVOO (8).

Coconut oil is another edible oil that has gained popularity recently. It is comprised of ∼85% SFAs and has a high total phenolic content (11.8–29.2 mg gallic acid equivalents/100 g oil) (10–12). According to dietary recommendations, including those by the 2015 Dietary Guidelines for Americans, heart healthy dietary patterns should be low in SFAs (3). However, because coconut oil contains ∼60% medium-chain fatty acids (carbon, C6:0–C12:0), a large proportion of which is lauric acid (C12:0), coconut oil is claimed by some to produce net health benefits (11–15). Ingestion of medium-chain TGs has been shown to lead to improved body composition, without adversely affecting cardiometabolic risk factors (16). However, the authors of a recent review concluded that there was no evidence that consumption of coconut oil should be viewed differently from consumption of other sources of SFAs with regard to dietary recommendations (15). They examined 21 research papers including 8 clinical trials and 13 observational studies published through 2013, and updated to include 1 additional eligible study published in 2015, that focused on the consumption of coconut oil, coconut milk, or coconut cream by humans and outcomes relevant to cardiovascular disease or related risk factors.

A controlled feeding study of 45 healthy young Malaysian adults tested coconut oil, palm olein oil, and virgin olive oil for 5 wk, incorporated into high-protein diets at two-thirds of the 30% total dietary calories provided by fat (17, 18). Compared with olive oil consumption, coconut oil raised mean fasting total-C, LDL-cholesterol, and HDL-cholesterol concentrations. There were no significant differences observed between the 3 diets in plasma total homocysteine, in the concentrations of inflammatory markers including TNF-α, IL-1β, IL-6, IL-8, high-sensitivity C-reactive protein (hs-CRP), and interferon-γ, or in thrombogenicity indexes. Recently, Harris et al. compared the consumption of high-oleic safflower oil with that of virgin coconut oil in 12 postmenopausal women in the United States (19). Virgin coconut oil significantly increased total-C, LDL cholesterol, and HDL cholesterol, whereas safflower oil did not significantly alter lipid values. Total-C and HDL-cholesterol concentrations were significantly different between the 2 test oils, but there were no clear treatment effects on inflammatory markers or other cytokines.

Few trials have examined the effects of coconut oil consumption in comparison with PUFA-rich oils such as corn oil (15). In a small study in 9 healthy males fed corn oil or coconut oil in mixed diets for 9 d, coconut oil significantly increased total-C, VLDL cholesterol, intermediate-density lipoprotein cholesterol + LDL cholesterol, HDL cholesterol, total TGs, and VLDL-TGs (20). The objectives of the present trial were to assess the effects of corn oil and coconut oil on lipoprotein lipids, glucose homeostasis, and hs-CRP (the most commonly used and best-validated clinical indicator of inflammation) in adult men and women with LDL-cholesterol concentrations above desirable levels.

## Methods

### 

#### Design

This was a randomized crossover preliminary investigation conducted at 2 clinical research sites: MB Clinical Research (Boca Raton, FL) and Great Lakes Clinical Trials (Chicago, IL). The study included 2 screening visits followed by two 4-wk test periods, each with clinic visits on days 0, 15, and 29, and with a 3-wk washout between the 2 test periods. Diet conditions were started in a manner that allowed premenopausal women to be tested in the follicular phase of the menstrual cycle, whenever possible. The study was conducted in accordance with Good Clinical Practice Guidelines, the Declaration of Helsinki (21), and the United States 21 Code of Federal Regulations. An institutional review board (Aspire IRB, Santee, CA) approved the protocol before initiation of the study, and subjects provided written informed consent before any study procedures were performed.

#### Subjects

Subjects included men and women 18–79 y of age, inclusive, with a BMI (kg/m^2^) of 18.5–34.9. They were required to have a fasting LDL-cholesterol concentration ≥115 mg/dL and ≤190 mg/dL, and a TG concentration ≤375 mg/dL at screening. To enroll in the study, subjects had to be willing to consume study-related foods and follow the dietary instructions, maintain their usual physical activity level, and have no plans to change their smoking habits (if a smoker) throughout the trial.

Persons were excluded from participation if they had atherosclerotic cardiovascular disease, uncontrolled hypertension, an abnormal laboratory test result of clinical significance, or a history or presence of any clinically important pulmonary, endocrine (including type 1 or 2 diabetes mellitus), chronic inflammatory, hepatic, renal, hematologic, immunologic, dermatologic, neurologic, psychiatric, or biliary disorder. A history of cancer in the prior 5 y (except nonmelanoma skin cancer) was also exclusionary. Individuals who had experienced a change in body weight of ±4.5 kg over the past 3 mo, or who had extreme dietary habits or had been diagnosed with an eating disorder, were not enrolled. Female subjects could not be pregnant, planning to be pregnant during the study period, or lactating. If of childbearing potential, female subjects had to commit to the use of a medically approved form of contraception throughout the study. Use of medications intended to alter the lipid profile or known to influence glucose metabolism, weight-loss drugs or programs, systemic corticosteroid drugs, unstable antihypertensive medications, or daily nonsteroidal anti-inflammatory drugs, other than low-dose aspirin, were excluded within 4 wk of screening and throughout the study. Use of foods or dietary supplements that might alter lipid metabolism was also excluded within 2 wk of screening and throughout the study. None of the subjects were taking fish oil or other omega-3 fatty acid supplements within 8 wk before the screening visit. If a subject had an active infection or had used antibiotics within 5 d of any clinic visit, he or she waited for at least 5 d after the infection resolved or antibiotic use was completed before attending the next clinic visit, and the length of that subject's test period was extended accordingly.

#### Study products and diet

Subjects were randomly assigned via a computer-generated randomization scheme to receive study products (muffins or rolls) containing corn oil during the first test period followed by coconut oil during the second test period, or vice versa. The estimated nutrient content of each study product is shown in **Supplemental Table 1**. Subjects were instructed to consume 4 study products per day for the duration of each 4-wk test period, and to record study product intake in a daily log. Consumption of the study products provided 4 tablespoons per day of corn oil or coconut oil, replacing the same amount of oil in the background habitual diet. Each tablespoon of corn oil or coconut oil was 13.6 g and provided 122 kcal. The Mifflin-St. Jeor Equation (22), with an adjustment for energy expended in physical activity assessed by the Stanford 7-day Physical Activity Recall Questionnaire (23), was utilized to calculate each subject's energy needs. Subjects received dietary counseling at baseline (with reinforcement at later clinic visits) that included instructions on maintaining habitual energy intake with a focus on how to substitute the study products for other foods in their diets in order to prevent excess energy intake. Compliance with study product consumption was recorded as the percentage of scheduled servings of study products consumed, evaluated by the study staff based on the number of study products given to the subject and the number of products returned to the clinic. A diary was used to double-check against the primary compliance calculation. Subjects were queried regarding discrepancies between the diary and calculation, when applicable.

Subjects also completed 3-d diet records on 2 weekdays and 1 weekend day before baseline and in the last week of each test period. A copy of the 3-d diet record collected at the end of the first test period was given to the subjects as a reminder of how study foods were incorporated, and subjects were encouraged to follow the same dietary pattern during the second test period. Each 3-d diet record was analyzed for nutrient content with the use of Food Processor Nutrition Analysis & Fitness Software (version 11.4, ESHA, Salem, OR).

#### Clinical assessments

Fasting (≥9 h, water only) plasma lipid profile (total-C, HDL cholesterol, non-HDL cholesterol, calculated LDL cholesterol, total-C:HDL cholesterol ratio, and TGs) and hs-CRP concentrations were measured at screening, baseline, and after 2 and 4 wk of each test period. An intravenous glucose tolerance test (IVGTT) was performed at the beginning of the first test period (baseline) and at the end of each test period (24–29). The following variables were calculated from fasting and IVGTT glucose and insulin concentrations: insulin sensitivity index (SI), acute insulin response to glucose (AIRg), disposition index (AIRg × SI), the fractional disappearance rate from 10 to 50 min (Kg), and homeostasis model assessments of β-cell function (HOMA%B) and insulin sensitivity (HOMA%S) (24–31). SI was calculated according to the formula described by Tura et al. (26), but without the use of a constant as a scaling factor. Subjects were instructed to avoid engaging in vigorous physical activity and to consume ≥150 g/d of carbohydrate during the 24-h period before each IVGTT. In addition, subjects were instructed to abstain from use of tobacco products for ≥1 h before and during the IVGTT.

Local laboratories, LabCorp in Boca Raton, FL and Swedish Covenant Hospital in Chicago, IL, performed general screening metabolic and hematology panel measurements. EDTA-coated tubes were used for plasma isolation. Fasting plasma lipids and hs-CRP and IVGTT glucose and insulin measurements were performed by Cleveland HeartLab, Inc., Cleveland, OH. Clinical chemistry analyses were conducted on a Cobas 6000 analyzer (Roche Diagnostics, Indianapolis, IN). The lipid panel was measured via photometric assays (32), hs-CRP was measured with the use of nephelometry (33), glucose was measured via an enzymatic assay (34), and insulin was measured via an electrochemiluminescence immunoassay (35).

Body weight and resting, seated blood pressures and heart rate were assessed at each clinic visit. Blood pressure was measured 3 times, separated by a minimum of 1 min, and all 3 values were averaged. Adverse events (AEs) were evaluated at each clinic visit by inquiring with an open-ended question about any changes in health status since the last visit or last health status query.

#### Statistical analyses

A sample size of 20 subjects was calculated to have 80% power to detect a difference of ≥6% in LDL cholesterol response between test conditions, assuming a pooled SD of 9% based on prior studies conducted by the investigators, and a 2-sided α of 0.05. This sample size would also be expected to provide ≥80% statistical power to detect moderate to large differences in responses of ≥0.67 SDs for additional outcome variables. Because this trial was considered a preliminary investigation, no adjustments were made for multiple comparisons in order to minimize the risk of type II statistical errors. A sample of 25 subjects was randomized to account for attrition. Statistical analyses were conducted through the use of SAS for Windows (version 9.3; SAS Institute, Cary, NC). All tests of significance were performed at α = 0.05, 2-sided.

Analyses of baseline and demographic characteristics were conducted for all subjects who were randomized into the study and consumed ≥1 dose of the study product. Two analyses were completed, 1 in an efficacy evaluable sample of 24 subjects who provided usable data for both diet conditions, and 1 in a per protocol sample of 23 subjects. Before completing statistical assessments of the outcome variables, a decision was made to exclude 1 subject from the per protocol analysis because of excessive weight gain during the first diet condition (5.7 kg) and because the subject underwent an influenza vaccination 1 d before the final visit in the second diet condition, both of which were believed to have the potential to confound the evaluation of the metabolic and inflammatory outcome variables.

The primary outcome variable was the percentage change in LDL cholesterol from baseline (the mean of screening and baseline values before the first test condition) to each test condition (mean of values from days 15 and 29 of each diet period). The other lipoprotein lipid, hs-CRP, and glucose metabolism parameters were secondary outcome variables. No evidence of statistically significant or clinically important carryover (treatment × sequence interaction) was evident, therefore data from both treatment sequences were pooled for the evaluation of the effects of corn oil and coconut oil on changes or percentage changes from baseline.

Comparisons between treatment sequence groups for baseline and demographic characteristics were assessed by ANOVA with treatment as fixed effect (for continuous variables) or by Fisher's exact (2-tail) test (for categoric variables). Outcome variables were assessed through the use of SAS Proc Mixed ANCOVA. Initial ANCOVA models contained terms for diet condition, sequence, period, and baseline value, with subject nested in sequence as a random effect. Models were reduced with a backward selection method until only significant terms or diet condition remained in the model. Assumptions of normality of residuals were investigated for each outcome. When there was a substantial departure from normality, assessed with the use of a quantile-quantile plot, values were ranked before running the final ANCOVA model. Within-treatment *P* values were generated by Wilcoxon’s Signed Rank test (lipid and hs-CRP variables) or the paired *t* test (body weight and vital signs), as appropriate. A post hoc comparison of the percentage of subjects who had an LDL cholesterol reduction ≥5% during the corn oil compared with coconut oil conditions was also conducted with the use of McNemar's test for changes in prevalence.

## Results

**FIGURE 1:**
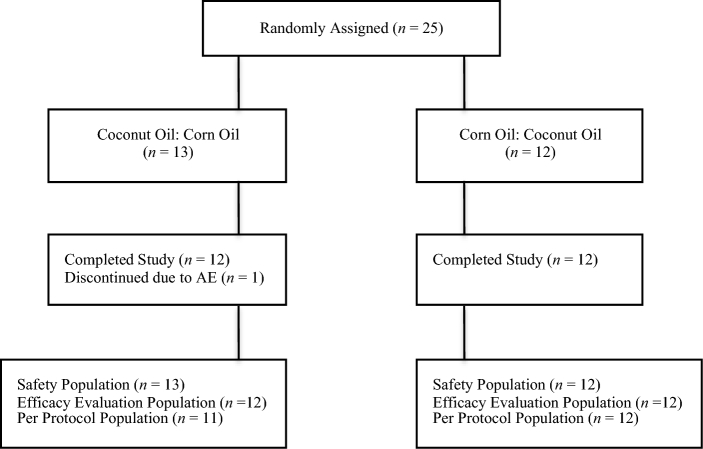
Flow diagram of subject disposition throughout the study. AE, adverse event.

Subject disposition is presented in **Figure 1**. A total of 25 subjects were randomly assigned. Of those, 1 subject (4.0%) discontinued the study because of an AE (intravenous site pain and redness) and was excluded from both the efficacy evaluable and per protocol analyses. An additional subject was excluded from the per protocol population as described previously. The results from the efficacy evaluable (*n* = 24) and per protocol (*n* = 23) samples were not notably different; results from the per protocol population are presented herein. There was 1 additional subject who did not have IVGTT data from either of the test periods because catheter patency could not be maintained, resulting in an IVGTT per protocol sample of 22 subjects.

There were no significant differences between the coconut oil-corn oil and corn oil-coconut oil sequences in any of the baseline or demographic characteristics. Subjects were 57% female and the majority were white (61%) and not of Hispanic/Latino ethnicity (78%) (**Table 1**). They had a mean ± SEM age of 45.2 ± 2.3 y and BMI of 27.7 ± 0.8.

**TABLE 1:** Demographic and baseline characteristics for all randomized subjects^1^

Characteristic	(*n* = 25)
Sex, *n* (%)	
Male	12 (48.0)
Female	13 (52.0)
Race, *n* (%)	
White	16 (64.0)
Black/African American	6 (24.0)
Asian or Pacific Islander	2 (8.0)
Did not specify	1 (4.0)
Ethnicity, *n* (%)	
Not Hispanic/Latino	20 (80.0)
Hispanic/Latino	2 (8.0)
Did not specify	3 (12.0)
Age, y	45.2 ± 2.3
Height, cm	171 ± 2.1
Weight, kg	81.6 ± 3.1
BMI, kg/m^2^	27.7 ± 0.8

^1^Values are means ± SEMs unless otherwise indicated. There were no significant differences between treatment sequences assessed by ANOVA with treatment as fixed effect for continuous variables and by Fisher's exact (2-tail) test for categoric variables.

Concentrations of lipoprotein lipids and hs-CRP at baseline and the percentage changes from baseline to the end of each test condition are shown in **Table 2**. The mean non-HDL cholesterol response was significantly different between corn oil (−3.0%) and coconut oil (+5.8%) conditions (*P* = 0.034). There were no other statistically significant differences in lipoprotein lipid or hs-CRP responses between test conditions, although the difference in total-C responses between corn oil (−0.5%) and coconut oil (+7.1%) conditions neared significance (*P* = 0.06), as did the difference in LDL cholesterol responses (−2.7% and +4.6% for corn oil and coconut oil periods, respectively, *P* = 0.06). The increase from baseline total-C for coconut oil was also statistically significant (*P* = 0.013 compared with baseline), as was the increase from baseline HDL cholesterol for both corn oil (5.4%; *P* = 0.007 compared with baseline) and coconut oil periods (6.5%; *P* < 0.001 compared with baseline). The change from baseline in the total-C:HDL cholesterol ratio with corn oil also neared statistical significance (−4.3%; *P* = 0.05). Significantly more subjects had an LDL cholesterol reduction ≥5% during the corn oil condition (10/23 subjects) than during the coconut oil condition (4/23 subjects) (*P* = 0.034).

**TABLE 2:** Concentrations of plasma lipoprotein lipids and hs-CRP at baseline and the changes from baseline after 4 wk consumption of corn oil or coconut oil study products in hypercholesterolemic adults in the per-protocol population^1^

Parameter	Baseline	Coconut oil, %∆ from baseline	Corn oil, %∆ from baseline
LDL-C, mg/dL	123 (105, 142)	4.6 (−2.5, 17.5)	−2.7 (−8.9, 11.5)
Non-HDL-C, mg/dL	144 (135, 161)	5.8 (−2.8, 11.1)	−3.0 (−6.9, 7.4)*
Total-C, mg/dL	188 (178, 215)	7.1 (−1.1, 13.1)^†^	−0.5 (−5.7, 9.7)
HDL-C, mg/dL	46.0 (38.5, 55.5)	6.5 (2.7, 17.8)^†^	5.4 (1.4, 10.3)^†^
Total-C:HDL-C	4.21 (3.65, 5.10)	−3.3 (−15.0, 2.8)	−4.3 (−11.7, 1.8)^†^
TG, mg/dL	92.5 (76.5, 136)	6.0 (−3.0, 13.2)	−2.1 (−9.7, 20.6)
hs-CRP, mg/L	1.55 (0.90, 4.00)	3.8 (−14.3, 41.7)	0.0 (−16.7, 33.8)

^1^Values are medians (IQRs), *n* = 23. *Different from coconut oil (*P* = 0.034) based on repeated-measures ANCOVA with subject included as a random effect; values were ranked before ANCOVA. ^†^Statistically significant %∆ from baseline (*P* ≤ 0.05) by Wilcoxon's Signed Rank test. HDL-C, high-density lipoprotein cholesterol; hs-CRP, high-sensitivity C-reactive protein; LDL-C, low-density lipoprotein cholesterol; non-HDL-C, non-high-density lipoprotein cholesterol; total-C, total cholesterol; %∆, percentage of change.

Although diet conditions were started in a manner to allow premenopausal women to be tested in the follicular phase of the menstrual cycle, a scheduling conflict prevented 1 subject from being tested during the follicular phase during the first treatment period. For the second treatment period, she was tested on the same day of her menstrual cycle as for the first treatment period. Both treatments were extended by a few days to maintain consistency. Exclusion of that subject from the analyses did not alter the findings (data not shown). Sensitivity analyses were also conducted to evaluate whether there was a larger difference between conditions when the lipoprotein lipid data from the last visit in each diet condition (day 29 only) were used to calculate responses rather than the mean of values from days 15 and 29. There was no evidence that the difference in responses was underestimated by use of the averaged values; however, the precision of the point estimates for the responses was lower (data not shown).

Results from the glucose homeostasis analyses are presented in **Table 3**. There were no significant differences between corn oil and coconut oil test conditions for any of the IVGTT variables measured including SI, disposition index, AIRg, Kg, HOMA%S, and HOMA%B.

**TABLE 3:** Plasma intravenous glucose tolerance test (IVGTT) variables at baseline and the changes from baseline after 4 wk consumption of corn oil or coconut oil study products in hypercholesterolemic adults in the IVGTT per protocol population^1^

Parameter	Baseline	Coconut oil, ∆ from baseline	Corn oil, ∆ from baseline
SI, × 10^−4^ min^−1^ × (μU/mL)^−1^	4.98 (3.04, 12.0)	−0.008 (−1.65, 1.85)	0.075 (−1.31, 2.28)
Disposition Index	0.299 (0.191, 0.353)	−0.019 (−0.110, 0.046)	−0.026 (−0.207, 0.164)
AIRg, μU/mL × min	368 (227, 851)	−48.1 (−178, 46.5)	−12.9 (−218, 146)
Kg, %/min	1.52 (1.22, 2.43)	−0.163 (−0.675, 0.245)	−0.141 (−0.616, 0.112)
HOMA%S	109 (64.8, 213)	0.7 (−25.0, 28.1)	−9.5 (−34.5, 35.9)
HOMA%B	90.0 (70.0, 117)	0.7 (−19.0, 11.0)	−6.7 (−22.3, 6.4)

^1^Values are medians (IQRs), *n* = 22. There were no significant differences between conditions based on repeated-measures ANCOVA with subject included as a random effect; values were ranked before ANCOVA. AIRg, acute insulin response to glucose; HOMA%B, homeostasis model assessment of β-cell function; HOMA%S, homeostasis model assessment of insulin sensitivity; IVGTT, intravenous glucose tolerance test; Kg, fractional disappearance rate from 10 to 50 min; SI, insulin sensitivity index; ∆, change.

Mean ± SEM compliance with consumption of study products in the per protocol sample was 98.0% ± 0.9% and 97.3% ± 0.7% for the corn oil and coconut oil conditions, respectively. Intakes of energy and selected nutrients at baseline and at the end of each test condition are shown in **Table 4**. Total energy intake increased during both test conditions from a baseline of 1950 kcal/d to 2549 kcal/d for corn oil and 2565 kcal/d for coconut oil. Increases from baseline are consistent with the known phenomenon of subjects underreporting energy intake on diet records (36, 37), combined with a more accurate assessment of energy intake from study foods than from food items in the habitual diet. Changes from baseline in dietary intakes were not significantly different between test conditions with the exception of SFA and unsaturated fatty acid (UFA) intakes (both *P* < 0.001 for differences between test conditions). Baseline SFA and UFA intakes were 11.6% and 24.1% of energy, respectively. Reflecting the SFA/UFA profiles of the study products, during the coconut oil test condition SFA intake increased to 25.1% of total energy, and UFA intake decreased to 19.4% of energy; during the corn oil test condition, SFA decreased to 10.7% of total energy and UFA increased to 33.7% of energy.

**TABLE 4:** Energy and nutrient intakes at baseline and after 4 wk consumption of corn oil or coconut oil study products in hypercholesterolemic adults in the per protocol population^1^

Parameter	Baseline	Coconut oil	Corn oil
Energy, kcal/d	1950 ± 117	2565 ± 98.0	2549 ± 94.4
Carbohydrate, % of energy	47.0 ± 2.3	41.4 ± 1.2	41.8 ± 1.5
Protein, % of energy	16.9 ± 0.9	14.6 ± 0.9	13.6 ± 0.7
Total fat, % of energy	35.7 ± 1.6	44.5 ± 0.8	44.4 ± 0.9
SFAs, % of energy	11.6 ± 0.7	25.1 ± 0.6	10.7 ± 0.4*
UFAs, % of energy	24.1 ± 1.2	19.4 ± 0.8	33.7 ± 0.8*
Dietary fiber, g/d	16.9 ± 1.1	16.8 ± 1.0	16.6 ± 1.3
Soluble dietary fiber, g/d	1.7 ± 0.3	1.2 ± 0.2	1.3 ± 0.2
Cholesterol, mg/d	294 ± 34.5	363 ± 31.2	339 ± 31.0
Sodium, g/d	2.8 ± 0.2	2.3 ± 0.2	2.4 ± 0.2
Calcium, g/d	0.7 ± 0.1	0.6 ± 0.1	0.6 ± 0.1

^1^Values are means ± SEMs, *n* = 23. *Different from coconut oil, *P* < 0.001, based on repeated-measures ANCOVA with subject included as a random effect. UFAs, unsaturated fatty acids.

Body weight and vital signs at baseline and changes from baseline during the test periods are shown in **Supplemental Table 2**. There were no significant differences between test conditions in the changes from baseline body weight or vital signs. After the corn oil condition mean body weight increased 0.6 kg (*P* = 0.007) and after the coconut oil condition mean body weight increased 0.2 kg (*P* = 0.42). After the coconut oil condition, subjects also had a significant increase from baseline in mean heart rate (2.8 bpm; *P* = 0.032). Eight (32%) and 7 (29%) subjects, in the coconut oil and corn oil conditions, respectively, reported ≥1 treatment-emergent AE. Most of the AEs were mild or moderate in nature, and none were serious. Six of the AEs during the coconut oil condition were deemed to be definitely, probably, or possibly related to treatment and/or the study procedures: diarrhea (*n* = 1), gastroenteritis (*n* = 1), gastrointestinal upset (*n* = 1), weight gain (*n* = 2), and intravenous site pain/redness (*n* = 1) which led to that subject's discontinuation from the study. Two of the AEs during the corn oil condition were deemed to be definitely, probably, or possibly related to treatment: constipation (*n* = 1) and weight gain (*n* = 1).

## Discussion

The results of this randomized crossover study indicate that, when incorporated into the habitual diet, consumption of muffins and rolls providing 4 tablespoons per day (∼54 g/d) of corn oil resulted in significantly reduced non-HDL-cholesterol concentrations (−3.0%) compared with consumption of similar products providing coconut oil (+5.8%) (*P* = 0.034) in adult men and women with above-desirable LDL-cholesterol levels. Non-HDL cholesterol reflects the amount of cholesterol carried within all atherogenic particles, including TG-rich VLDLs and remnant lipoproteins in addition to LDL particles (38). LDL-cholesterol and total-C concentrations also tended to increase with coconut oil consumption (+4.6% and +7.1%, respectively) compared with corn oil consumption (−2.7% and −0.5%, respectively). However, the differences between those responses did not quite reach statistical significance (*P* = 0.06 for both). HDL cholesterol rose significantly (*P* < 0.05) with consumption of both coconut oil and corn oil (6.5% and 5.4%, respectively). These results are consistent with those from other studies showing that coconut oil, which is high in SFAs, generally raises total-C and LDL cholesterol to a greater extent than unsaturated plant oils (15), and support the 2015 Dietary Guidelines for Americans that recommend dietary patterns low in SFAs and richer in UFAs, especially PUFAs, for the reduction of coronary heart disease risk (3). Although coconut oil has been shown to increase LDL cholesterol, due to a concomitant increase in HDL cholesterol that also generally occurs, the total-C:HDL cholesterol ratio often declines (39, 40). In the present study the total-C:HDL cholesterol ratio decreased by 3.3% with coconut oil and 4.3% with corn oil. Only the corn oil–induced total-C:HDL cholesterol reduction approached statistical significance (*P* = 0.05).

Although coconut oil has recently been promoted in the commercial press as healthy, the results from several clinical trials, including the present findings, suggest that, with regard to lipoprotein lipid effects, coconut oil is not an optimal choice (15). The healthy reputation stems, in part, from the fact that about half of the fatty acid content of coconut oil is lauric acid (C12:0), which is classified by some sources as a medium-chain fatty acid (41). However, some experts argue that C12:0 has characteristics more similar to long-chain fatty acids than to the C6:0–C10:0 medium-chain fatty acids (42–44). Coconut oil contains ∼14% C6:0–C10:0 fatty acids (41). Medium-chain TGs are more water-soluble and are more easily absorbed than long-chain TGs. After their absorption they are transported directly into the liver and undergo β-oxidation preferentially compared with longer-chain fatty acids. Medium-chain TGs, as a replacement of long-chain TGs, have been shown in animal studies and trials in humans to increase energy expenditure, increase satiety, reduce body weight, and reduce percentage of body fat (42, 45). However, there is some controversy over whether medium-chain TGs composed primarily of lauric acid, such as those in coconut oil, have the same thermogenic effects and fat oxidation–enhancing abilities as those that contain mainly caprylic and capric fatty acids (39, 44, 46–48).

Besides the differences in fatty acid composition, both coconut oil and corn oil contain other components with potentially bioactive properties. Corn oil contains 534 mg phytosterols/54 g (4 tablespoons) of oil, which is likely responsible for part of its cholesterol-lowering effects (1, 8, 49, 50). Coconut oil, particularly virgin coconut oil, also contains some phytosterols (38.6 mg total sterols/54 g crude coconut oil [4 tablespoons]) and bioactive compounds such as tocotrienols, tocopherols, and polyphenols (total phenolic content of 11.8–29.2 mg gallic acid equivalents/100 g oil) (10–12, 51, 52).

The magnitude of atherogenic cholesterol–lowering with corn oil in the present study was lower than had been anticipated, based on results from a previous controlled feeding trial in which consumption of foods made with corn oil was compared with consumption of foods made with EVOO (8, 9). In that study, LDL cholesterol was reduced by 10.9% in the corn oil condition compared with 3.5% in the EVOO condition. Corresponding values for total-C were reductions of 8.2% (corn oil) compared with 1.8% (EVOO), and for non-HDL cholesterol, reductions of 9.3% (corn oil) compared with 1.6% (EVOO) (all *P* < 0.001 between conditions). The total-C:HDL cholesterol ratio was decreased by 4.4% with corn oil, which was very similar to the reduction of 4.3% found for corn oil in the present trial. The smaller atherogenic-cholesterol–lowering responses to corn oil detected in this trial, compared with the previous controlled trial, might be attributable to the background habitual diet. The subjects’ habitual diet (including study products) during the 2 test periods was higher in SFAs (corn oil, 10.7%; coconut oil, 25.1%) and cholesterol (corn oil, 339 mg/d; coconut oil, 363 mg/d) than the controlled diet provided to subjects in the previous study (<10% SFA, cholesterol <300 mg/d).

Subjects received dietary counseling regarding the incorporation of the study products into their habitual diets, with an emphasis on food substitutions, and a goal of maintaining habitual energy intake. Mean body weight increased by 0.6 kg in the corn oil condition, compared with a 0.2 kg mean increase in the coconut oil condition; there was no significant difference in body weight change between test conditions. According to 3-d diet records, the total energy intake was higher during both test conditions than at baseline, but to a similar degree.

There is a substantial body of evidence from studies investigating the effects of edible oils on lipoprotein lipids, but fewer studies have investigated the effects of these oils on other cardiometabolic risk factors including markers of inflammation and insulin sensitivity. In the present trial, no difference in hs-CRP responses was observed between the coconut oil (+3.8%) and corn oil conditions (0.0%). This finding is consistent with results from other examinations showing no effects of coconut oil, corn oil, or other high–ω-6 PUFA oils on inflammatory markers (15, 17, 18, 53, 54). Similarly, the results for markers of glucose homeostasis showed that there were no significant differences in measures of insulin sensitivity and other markers between the corn oil and coconut oil conditions, and no significant changes from the baseline values within treatment conditions. Additional randomized controlled clinical trials with larger numbers of subjects are needed to further examine the potential effects of PUFA- and phytosterol-rich compared with SFA-rich food oils on these parameters.

The results from the present trial indicate that, when incorporated into the habitual diet, consumption of foods providing 4 tablespoons per day (∼54 g/d) of corn oil, which contains a higher quantity of plant sterols than other cooking oils and is rich in PUFAs, reduced non-HDL cholesterol compared with coconut oil (high in SFAs) in men and women with above-desirable levels of circulating cholesterol. No differences were observed in responses for hs-CRP (a marker for inflammation) or indexes of glucose homeostasis, including fasting and dynamic indexes of insulin sensitivity and pancreatic β-cell function.

## Supplementary Material

Supplemental File

## Notes

Supported by ACH Food Companies, Inc., Oakbrook Terrace, IL.

Supplemental Tables 1 and 2 are available from the “Supplementary data” link in the online posting of the article and from the same link in the online table of contents at https://academic.oup.com/jn/.

Author disclosures: KCM, WH, MRD, MB, and MAB received research funding from ACH Food Companies, Inc. MEC and FE are employees of ACH Food Companies, Inc.

**Abbreviations used**
AE: adverse event
AIRg: acute insulin response to glucose
EVOO: extra-virgin olive oil
HOMA%B: homeostasis model assessment of β-cell function
HOMA%S: homeostasis model assessment of insulin sensitivity
hs-CRP: high-sensitivity C-reactive protein
IVGTT: intravenous glucose tolerance test
Kg: fractional disappearance rate
SI: insulin sensitivity index
total-C: total cholesterol
UFA: unsaturated fatty acid

## References

[1] Ostlund RE Jr, Racette SB, Okeke A, Stenson WF Phytosterols that are naturally present in commercial corn oil significantly reduce cholesterol absorption in humans. Am J Clin Nutr 2002;75:1000–4. 12036805 10.1093/ajcn/75.6.1000

[2] Mozaffarian D, Micha R, Wallace S Effects on coronary heart disease of increasing polyunsaturated fat in place of saturated fat: a systematic review and meta-analysis of randomized controlled trials. PLoS Med 2010;7:e1000252. 20351774 10.1371/journal.pmed.1000252 PMC2843598

[3] US Department of Health and Human Services and US Department of Agriculture. 2015–2020 Dietary Guidelines for Americans (DGA). 8th ed December 2015 [cited Sep 22 2016]. Available from: http://health.gov/dietaryguidelines/2015/guidelines/.

[4] US Department of Agriculture, Agricultural Research Service. USDA Food Composition Databases. Corn oil [Internet] 2018 [cited 2018 Apr 14]. Available from: https://ndb.nal.usda.gov/ndb/search/list?SYNCHRONIZER_TOKEN=04ed0f64-a9e7-4fc9-918b-8b82a693d120&SYNCHRONIZER_URI=%2Fndb%2Fsearch%2Flist&qt=&ds=&qlookup = corn+oil&manu=.

[5] Food and Drug Administration (FDA). Qualified health claims: letter of enforcement discretion – corn oil and corn oil-containing products and a reduced risk of heart disease (Docket No. 2006P-0.43) [Internet]. 2007 [cited 2018 Apr 14]. Available from: http://wayback.archive-it.org/7993/20171114183735/https://www.fda.gov/Food/IngredientsPackagingLabeling/LabelingNutrition/ucm072956.htm.

[6] Lichtenstein AH, Ausman LM, Carrasco W, Jenner JL, Gualitieri LJ, Goldin BR, Ordovas JM, Schaefer EJ Effects of canola, corn, and olive oils on fasting and postprandial plasma lipoproteins in humans as part of a National Cholesterol Education Program Step 2 diet. Arterioscler Thromb 1993;13:1533–42. 8399091 10.1161/01.atv.13.10.1533

[7] Wagner KH, Tomasch R, Elmadfa I Impact of diets containing corn oil or olive/sunflower oil mixture on the human plasma and lipoprotein lipid metabolism. Eur J Nutr 2001;40:161–7. 11905957 10.1007/s003940170004

[8] Maki KC, Lawless AL, Kelley KM, Kaden VN, Geiger CJ, Dicklin MR Corn oil improves the plasma lipoprotein lipid profile compared with extra-virgin olive oil consumption in men and women with elevated cholesterol: results from a randomized controlled feeding trial. J Clin Lipidol 2015;9:49–57. 25670360 10.1016/j.jacl.2014.10.006

[9] Maki KC, Lawless AL, Kelley KM, Kaden VN, Geiger CJ, Palacios OM, Dicklin MR Corn oil intake favorably impacts lipoprotein cholesterol, apolipoprotein and lipoprotein particle levels compared with extra-virgin olive oil. Eur J Clin Nutr 2017;71:33–8. 27677368 10.1038/ejcn.2016.169

[10] Pehowich DJ, Gomes AV, Barnes JA Fatty acid composition and possible health effects of coconut constituents. West Indian Med J 2000;49:129–33. 10948851

[11] Amarasiri WA, Dissanayake AS Coconut fats. Ceylon Med J 2006;51:47–51. 17180807 10.4038/cmj.v51i2.1351

[12] Babu AS, Veluswamy SK, Arena R, Guazzi M, Lavie CJ Virgin coconut oil and its potential cardioprotective effects. Postgrad Med 2014;126:76–83. 10.3810/pgm.2014.11.2835 25387216

[13] Denke MA, Grundy SM Comparison of effects of lauric acid and palmitic acid on plasma lipids and lipoproteins. Am J Clin Nutr 1992;56:895–8. 1415008 10.1093/ajcn/56.5.895

[14] Kris-Etherton PM, Yu S Individual fatty acid effects on plasma lipids and lipoproteins: human studies. Am J Clin Nutr 1997;65(Suppl):1628S–44. 9129503 10.1093/ajcn/65.5.1628S

[15] Eyres L, Eyres MF, Chisholm A, Brown RC Coconut oil consumption and cardiovascular risk factors in humans. Nutr Rev 2016;74:267–80. 26946252 10.1093/nutrit/nuw002 PMC4892314

[16] Bhavsar N, St-Onge MP The diverse nature of saturated fats and the case of medium-chain triglycerides: how one recommendation may not fit all. Curr Opin Clin Nutr Metab Care 2016;19:81–7. 26727347 10.1097/MCO.0000000000000249

[17] Voon PT, Ng TK, Lee VK, Nesaretnam K Diets high in palmitic acid (16:0), lauric and myristic acids (12:0 + 14:0), or oleic acid (18:1) do not alter postprandial or fasting plasma homocysteine and inflammatory markers in healthy Malaysian adults. Am J Clin Nutr 2011;94:1451–7. 22030224 10.3945/ajcn.111.020107

[18] Voon PT, Ng TK, Lee VK, Nesarethnam K Virgin olive oil, palm olein and coconut oil diets do not raise cell adhesion molecules and thrombogenicity indices in healthy Malaysian adults. Eur J Clin Nutr 2015;69:712–16. 25804278 10.1038/ejcn.2015.26

[19] Harris M, Hutchins A, Fryda L The impact of virgin coconut oil and high-oleic safflower oil on body composition, lipids, and inflammatory markers in postmenopausal women. J Med Food 2017;20:345–51. 28277823 10.1089/jmf.2016.0114

[20] Fisher EA, Blum CB, Zannis VI, Breslow JL Independent effects of dietary saturated fat and cholesterol on plasma lipids, lipoproteins, and apolipoprotein E. J Lipid Res 1983;24:1039–48. 6631232

[21] World Medical Association. World Medical Association Declaration of Helsinki ethical principles for medical research involving human subjects. JAMA 2013;310:2191–4. 24141714 10.1001/jama.2013.281053

[22] Mifflin MD, St Jeor ST, Hill LA, Scott BJ, Daughtery SA, Koh YO A new predictive equation for resting energy expenditure in healthy individuals. Am J Clin Nutr 1990;51:241–7. 2305711 10.1093/ajcn/51.2.241

[23] Blair SN, Haskell WL, Ho P, Paffenbarger RS Jr, Vranizan KM, Farquhar JW, Wood PD Assessment of habitual physical activity by a seven-day recall in a community survey and controlled experiments. Am J Epidemiol 1985;122:794–804. 3876763 10.1093/oxfordjournals.aje.a114163

[24] Galvin P, Ward G, Walters J, Pestell R, Koschmann M, Vaag A, Martin I, Best JD, Alford F A simple method for quantitation of insulin sensitivity and insulin release from an intravenous glucose tolerance test. Diabet Med 1992;9:921–8. 1478037 10.1111/j.1464-5491.1992.tb01732.x

[25] Anderson RL, Hamman RF, Savage PJ, Saad MF, Laws A, Kades WW, Sands RE, Cefalu W Exploration of simple insulin sensitivity measures derived from frequently sampled intravenous glucose tolerance (FSIGT) tests. The Insulin Resistance Atherosclerosis Study. Am J Epidemiol 1995;142:724–32. 7572943 10.1093/aje/142.7.724

[26] Tura A, Sbrignadello S, Succurro E, Groop L, Sesti G, Pacini G An empirical index of insulin sensitivity from short IVGTT: validation against the minimal model and glucose clamp indices in patients with different clinical characteristics. Diabetologia 2010;53:144–52. 19876614 10.1007/s00125-009-1547-9

[27] Maki KC, Cham CM, Huebner M, Palacios OM Evaluation of an insulin sensitivity index from the short intravenous glucose tolerance test. FASEB J 2016;30:Ib287.

[28] Maki KC, Nieman KM, Cook CM, Huebner M, Baum SJ Evaluation of an insulin sensitivity index from the short intravenous glucose tolerance test for detecting response to a dietary intervention. FASEB J 2016;30:lb288.

[29] Palacios OM, Nieman KM, Cook CM, Maki KC Evaluation of a reduced sampling schedule for assessment of pancreatic beta-cell function with the intravenous glucose tolerance test during a dietary intervention. FASEB J 2017;31:Ib284.

[30] Wallace TM, Levy JC, Matthews DR Use and abuse of HOMA modeling. Diabetes Care 2004;27:1487–95. 15161807 10.2337/diacare.27.6.1487

[31] University of Oxford Diabetes Trials Unit, The Oxford Centre for Diabetes, Endocrinology and Metabolism. HOMA calculator [Internet]. 2018 [cited 2018 Apr 14]. Available from: http://www.dtu.ox.ac.uk/homacalculator/.

[32] Cleveland Heart Lab. Lipid panel [Internet]. 2013 [cited 2018 April 14]. Available from: http://www.clevelandheartlab.com/tests/lipid-panel/.

[33] Cleveland Heart Lab. C-Reactive Protein (CRP) [Internet]. 2013 [cited 2018 April 14]. Available from: http://www.clevelandheartlab.com/tests/c-reactive-protein/.

[34] Cleveland Heart Lab. Glucose [Internet]. 2013 [cited 2018 April 14]. Available from: http://www.clevelandheartlab.com/tests/glucose/.

[35] Cleveland Heart Lab. Insulin, total [Internet]. 2013 [cited 2018 April 14]. Available from: http://www.clevelandheartlab.com/tests/insulin-total/.

[36] Johnson RK Dietary intake—how do we measure what people are *really* eating? Obesity 2002;10:63S–8. 10.1038/oby.2002.192 12446861

[37] Maki KC, Palacios OM, Lindner E, Nieman KM, Bell M, Sorce J Replacement of refined starches and added sugars with egg protein and unsaturated fats increases insulin sensitivity and lowers triglycerides in overweight or obese adults with elevated triglycerides. J Nutr 2017;147:1267–74. 28515160 10.3945/jn.117.248641

[38] Jacobson TA, Ito MK, Maki KC, Orringer CE, Bays HE, Jones PH, McKenney JM, Grundy SM, Gill EA, Wild RA National Lipid Association recommendations for patient-centered management of dyslipidemia: part 1—full report. J Clin Lipidol 2015;9:129–69. 25911072 10.1016/j.jacl.2015.02.003

[39] McCarty MF, DiNicolantonio JJ Lauric acid-rich medium-chain triglycerides can substitute for other oils in cooking applications and may have limited pathogenicity. Open Heart 2016;3:e000467. 27547436 10.1136/openhrt-2016-000467 PMC4975867

[40] Chinwong S, Chinwong D, Mangklabruks A Daily consumption of virgin coconut oil increases high-density lipoprotein cholesterol levels in healthy volunteers: a randomized crossover trial. Evid Based Complement Alternat Med 2017;2017:7251562. 29387131 10.1155/2017/7251562 PMC5745680

[41] Orsavova J, Misurcova L, Ambrozova JV, Vicha R, Mlcek J Fatty acid composition of vegetable oils and its contribution to dietary intake and dependence of cardiovascular mortality on dietary intake of fatty acids. Int J Mol Sci 2015;16:12871–90. 26057750 10.3390/ijms160612871 PMC4490476

[42] St-Onge MP, Jones JH Physiological effects of medium-chain triglycerides: potential agents in the prevention of obesity. J Nutr 2002;132:329–32. 11880549 10.1093/jn/132.3.329

[43] Marten B, Pfeuffer M, Schrezenmeir J Medium-chain triglycerides. Int Dairy J 2006;16:1374–82.

[44] LaBarrie J, St-Onge MP A coconut oil-rich meal does not enhance thermogenesis compared to corn oil in a randomized trial in obese adolescents. Insights Nutr Metab 2017;1:30–6. 28758166 PMC5531289

[45] Mumme K, Stonehouse W Effects of medium-chain triglycerides on weight loss and body composition: a meta-analysis of randomized controlled trials. J Acad Nutr Diet 2015;115:249–63. 25636220 10.1016/j.jand.2014.10.022

[46] DeLany JP, Windhauser MM, Champagne CM, Bray GA Differential oxidation of individual dietary fatty acids in humans. Am J Clin Nutr 2000;72:905–11. 11010930 10.1093/ajcn/72.4.905

[47] Aoyama T, Nosaka N, Kasai M Research on the nutritional characteristics of medium-chain fatty acids. J Med Invest 2007;54:385–8. 17878693 10.2152/jmi.54.385

[48] St-Onge MP, Mayrsohn B, O'Keeffe M, Kissileff HR, Choudhury AR, Laferrere B Impact of medium and long chain triglycerides consumption on appetite and food intake in overweight men. Eur J Clin Nutr 2014;68:1134–40. 25074387 10.1038/ejcn.2014.145 PMC4192077

[49] Howell TJ, MacDougall DE, Jones LJH Phytosterols partially explain differences in cholesterol metabolism caused by corn or olive oil feeding. J Lipid Res 1998;39:892–900. 9555952

[50] Silbernagel G, Genser B, Nestel P, Marz W Plant sterols and atherosclerosis. Curr Opin Lipidol 2013;24:12–17. 23165086 10.1097/MOL.0b013e32835b6271

[51] Forcades M, Verhe R, Dewettinck K, Huyghebaert A, De Greyt W Analysis of free and esterified sterols in vegetable oils. J Am Oil Chem Soc 2002;79:117–22.

[52] Illam SP, Navarayanankutty A, Raghavamenon AC Polyphenols of virgin coconut oil prevent pro-oxidant mediated cell death. Toxicol Mech Methods 2017;27:442–50. 28412883 10.1080/15376516.2017.1320458

[53] Johnson GH, Fritsche K Effect of dietary linoleic acid on markers of inflammation in healthy persons: a systematic review of randomized controlled trials. J Acad Nutr Diet 2012;112:1029–41. 22889633 10.1016/j.jand.2012.03.029

[54] Maki KC, Eren F, Cassens ME, Dicklin MR, Davidson MH Omega-6 polyunsaturated fatty acids and cardiometabolic health: current evidence, controversies, and research gaps. Adv Nutr 2018; In press. 10.1093/advances/nmy038 PMC6247292 30184091

