# Supplementary material for: Corn Oil Lowers Plasma Cholesterol Compared with Coconut Oil in Adults with Above-Desirable Levels of Cholesterol in a Randomized Crossover Trial
Source: J Nutr. 2018 Sep 10;148(10):1556–63. doi: 10.1093/jn/nxy156 (PMC6168703; doi:10.1093/jn/nxy156)
Supplement: Supplemental File [file nxy156_supplemental_file.docx]

**Supplementary Data**

**Supplemental Table 1.** Estimated nutrient content of each study product^1^

|  | **Energy (kcal)** | **CHO (g)** | **PRO (g)** | **Fat (g)** | **SFA (g)** | **MUFA (g)** | **PUFA (g)** | **CHOL (mg)** | **Fiber (g)** |
| --- | --- | --- | --- | --- | --- | --- | --- | --- | --- |
| **Corn Oil Study Products** | | | | | | | | | |
| Banana muffin | 230 | 20.5 | 2.3 | 15.4 | 2.1 | 4.3 | 8.2 | 24.1 | 0.5 |
| Chocolate muffin | 229 | 20.5 | 2.2 | 15.6 | 2.3 | 4.4 | 8.2 | 24.1 | 0.8 |
| Lemon-poppyseed muffin | 231 | 20.3 | 2.2 | 15.6 | 2.1 | 4.3 | 8.3 | 24.1 | 0.5 |
| Italian herb roll | 265 | 27.4 | 4.8 | 14.9 | 2.0 | 4.0 | 8.1 | 0.3 | 0.9 |
| Onion roll | 265 | 27.7 | 4.9 | 14.8 | 1.9 | 4.0 | 8.0 | 0.3 | 1.0 |
| Plain roll | 265 | 27.4 | 4.8 | 14.9 | 2.0 | 4.0 | 8.1 | 0.3 | 0.9 |
| **Coconut Oil Study Products** | | | | | | | | | |
| Banana muffin | 225 | 20.5 | 2.3 | 15.4 | 12.6 | 1.2 | 0.6 | 24.1 | 0.5 |
| Chocolate muffin | 224 | 20.5 | 2.2 | 15.6 | 12.8 | 1.3 | 0.6 | 24.1 | 0.8 |
| Lemon poppyseed muffin | 226 | 20.3 | 2.2 | 15.6 | 12.6 | 1.2 | 0.8 | 24.1 | 0.5 |
| Italian herb roll | 260 | 27.4 | 4.8 | 14.9 | 12.5 | 0.9 | 0.5 | 0.3 | 0.9 |
| Onion roll | 260 | 27.7 | 4.9 | 14.8 | 12.3 | 0.9 | 0.6 | 0.3 | 1.0 |
| Plain roll | 260 | 27.4 | 4.8 | 14.9 | 12.5 | 0.9 | 0.5 | 0.3 | 0.9 |

^1^CHO, carbohydrate; CHOL, cholesterol; MUFA, monounsaturated fatty acids; PRO, protein; PUFA, polyunsaturated fatty acids; SFA, saturated fatty acids

**Supplemental Table 2.** Body weight and vital signs at baseline and the changes from baseline after 4 weeks consumption of corn oil or coconut oil study products in hypercholesterolemic adults in the per protocol population^1^

| **Parameter** | **Baseline** | **Coconut Oil,**  **∆ from baseline** | **Corn Oil,**  **∆ from baseline** |
| --- | --- | --- | --- |
| Body Weight, kg | 81.2 ± 3.3 | 0.2 ± 0.3 | 0.6 ± 0.2^*^ |
| Systolic Blood Pressure, mm Hg | 116 ± 2.2 | 1.4 ± 1.6 | 0.3 ± 1.1 |
| Diastolic Blood Pressure, mm Hg | 76.0 ± 1.1 | -0.6 ± 1.1 | -0.5 ± 0.9 |
| Heart Rate, bpm | 68.4 ± 1.4 | 2.8 ± 1.2^*^ | 1.2 ± 1.1 |

^1^Data are presented as means ± SEs, *n* = 23. No significant difference between conditions based on repeated measures analysis of covariance with subject included as a random effect. ^*^Statistically significant ∆ from baseline (*P* < 0.05) by the paired t-test. ∆, change.
